# High-titer human packaging cells producing γ-retroviral vectors pseudotyped with engineered baboon endogenous retrovirus (BaEV) envelope proteins

**DOI:** 10.3389/fmmed.2026.1888550

**Published:** 2026-08-19

**Authors:** Angelina Hallik, Mona Pißarreck, Mats Jürgen Grummel, Yasemin van Heuvel, Jörn Stitz

**Affiliations:** 1 Research Group Medical Biotechnology & Bioengineering, Faculty of Applied Natural Sciences, TH Köln - University of Applied Sciences, Leverkusen, Germany; 2 Institute of Biology, University of Siegen, Siegen, Germany

**Keywords:** baboon endogenous retrovirus, cell line development, cell therapy, envelope protein, gene therapy, human packaging cell, murine leukemia virus pseudotype vector, retroviral vector

## Abstract

Lentiviral, α-retroviral, and γ-retroviral vector particles pseudotyped with envelope (Env) proteins of baboon endogenous retrovirus (BaEV) were shown to mediate highly efficient gene transfer into a range of primary human hematopoietic cell types in gene therapeutic strategies. However, the fusogenicity of utilized Env proteins mediated syncytia formation, hindering the establishment of stable viral packaging cell lines (VPCLs). Consequently, we used another BaEV Env variant, termed BaEV-TR—previously reported not to induce cell-to-cell fusion—and assessed its utility in stable producer cell line development. The absence of syncytia formation was reconfirmed in human 293T and HT1080 cell lines. Using HT1080 host cells, the stable VPCL HT/BaEV-TR was established. Conducting a Western blot analysis, BaEV-TR protein incorporation into vector particles was readily detected. Murine leukemia virus (MLV) pseudotype vectors efficiently transduced a variety of human target cell lines, reaching high vector titers of more than 3.0 × 10^6^ transducing units per mL (TU/mL) in 293T target cells. In summary, this proof-of-concept study underlines the utility of the Env variant BaEV-TR for the establishment of stable pseudotype VPCLs and the utilization of HT1080 cells as an alternative to frequently used 293T host cells.

## 1 Introduction

Vectors derived from the murine leukemia virus (MLV) play a pivotal role in gene therapeutic strategies. Therapeutic applications include monogenetic diseases of different hematopoietic cells, such as adenosine deaminase-deficient severe combined immunodeficiency (ADA-SCID) and Wiskott–Aldrich syndrome (WAS; Jaballah et al., 2025). In addition and particularly within the last 2 decades, γ-retroviral vectors were utilized to transduce chimeric antigen receptor (CAR) genes in cells of the immune system such as T helper, natural killer (NK) cells, and cytotoxic lymphocytes to target a variety of cancer types in CAR cell therapies (Yin and Wei, 2025). Today’s approved CAR therapies use MLV vectors pseudotyped with the wild-type (wt) envelope (Env) proteins of gibbon ape leukemia virus (GaLV), produced by the stable murine viral packaging cell line (VPCL) PG13 (Miller et al., 1991). Alternatively, Env proteins derived from the feline endogenous retrovirus RD114 can be used to generate MLV pseudotype vectors, achieving similar or slightly improved gene transfer efficiencies into human hematopoietic cells (Ghani et al., 2009).

Like RD114, the Env proteins of a closely related virus, baboon endogenous retrovirus (BaEV), recruit the sodium-dependent neutral amino acid transporter type 2 (ASCT2) as a receptor to facilitate cell entry (Tailor et al., 1999; Rasko et al., 1999). Moreover, BaEV uses ASCT1 as an auxiliary receptor (Marin et al., 2000). Lentiviral, α-retroviral, and γ-retroviral vector particles pseudotyped with BaEV Env proteins revealed highly efficient gene transfer into a variety of primary human hematopoietic cell types, outperforming the utilization of GaLV and RD114 Env proteins (Girard-Gagnepain et al., 2014; Ciulean et al., 2023; Klatt et al., 2024; Zhao et al., 2025). In these studies, wt BaEV Env and BaEVΔR Env proteins were utilized for pseudotyping. During particle maturation, mediated by the viral protease of γ-retroviruses, the processing of the cytoplasmic domain of the envelope proteins results in the cleavage of the R-peptide, which activates the fusogenicity of the Env proteins (Bobkova et al., 2002). BaEVΔR Env proteins are highly fusogenic and can be recombinantly expressed from engineered truncated *env* genes, which omit the proteolytic processing during particle maturation and, therefore, yield advanced titers when pseudotype vectors are generated using transient transfection (Girard-Gagnepain et al., 2014). However, when stable viral packaging cell lines (VPCLs) were established, both wt and ΔR BaEV Env proteins mediated cytotoxicity by the formation of syncytia, requiring the knockout of the cognate receptors ASCT1 and 2 (Klatt et al., 2024; Zhao et al., 2025).

In the past decades, retroviral envelope protein variants have frequently been generated with modified cytoplasmic domains to enhance pseudotype vector titers. Most commonly, the cytoplasmic tail was substituted with the homologous domain of MLV, first described by Lindemann et al. (1997), using foamy virus Env protein variants. These chimeric Env variants are often named after the donor virus, followed by the abbreviation TR, where T stands for the (cytoplasmic) tail and R for the R-peptide. Examples include GaLV-TR and RD114-TR (Sandrin et al., 2002; Sandrin et al., 2004; Di Nunzio et al., 2007; Kim et al., 2010; Boudeffa et al., 2019; Müller et al., 2020). In addition to enabling efficient R-peptide processing by the viral protease, the MLV R-peptide mediates interaction with the Gag core protein, resulting in efficient recruitment and incorporation of Env proteins during assembly and budding (Januszeski et al., 1997; Aguilar et al., 2003; Tschorn et al., 2022).

BaEV-TR Env proteins were reported to facilitate high-titer lentiviral pseudotype vector preparations but did not induce visible formation of large syncytia in transiently transfected 293T cells (Girard-Gagnepain et al., 2014). We thus first reconfirmed these findings in two different human cell lines and subsequently utilized BaEV-TR in a proof-of-concept (POC) study to establish a human MLV-based VPCL. The incorporation of Env proteins into γ-retroviral vector particles and, thus, the ability of the VPCL to mediate efficient formation of pseudotype vectors were assessed, and vector-mediated gene transfer efficiencies into a variety of human target cell lines were examined in titration experiments.

## 2 Article types

This is a brief research report.

## 3 Materials and methods

### 3.1 Cells

Human embryonic kidney 293T cells (ATCC CRL-3216) and human fibrosarcoma HT1080 cells (ATCC CCL-121) and their recombinant derivatives were grown in high-glucose (4.5 g/L) Dulbecco’s modified Eagle medium (DMEM; Gibco BRL, Germany) supplemented with 2 mM L-glutamine and 10% fetal bovine serum (FBS; Gibco BRL, Germany). Jurkat T lymphoblasts (clone E6-1; ATCC TIB-152), pro-monocytic U937 (ATCC CRL-1593.2), K562 lymphoblasts (ATCC CCL-243), and acute promyelocytic leukemia NB4 cells (Cytion cat. # 300299; Lanotte et al., 1991) were cultivated in RPMI 1640 medium (Gibco BRL, Germany) supplemented with 2 mM L-glutamine, 4.5 g/L glucose, 2.383 g/L HEPES buffer, 1.5 g/L sodium bicarbonate, 110 mg/L sodium pyruvate, and 10% FBS. Cells were expanded at 37 °C in a humidified atmosphere at 5% CO_2_. For passaging, adherent cells were detached using TrypLE (Gibco BRL, Germany).

### 3.2 Construction of expression and transposon vectors

**FIGURE 1:**
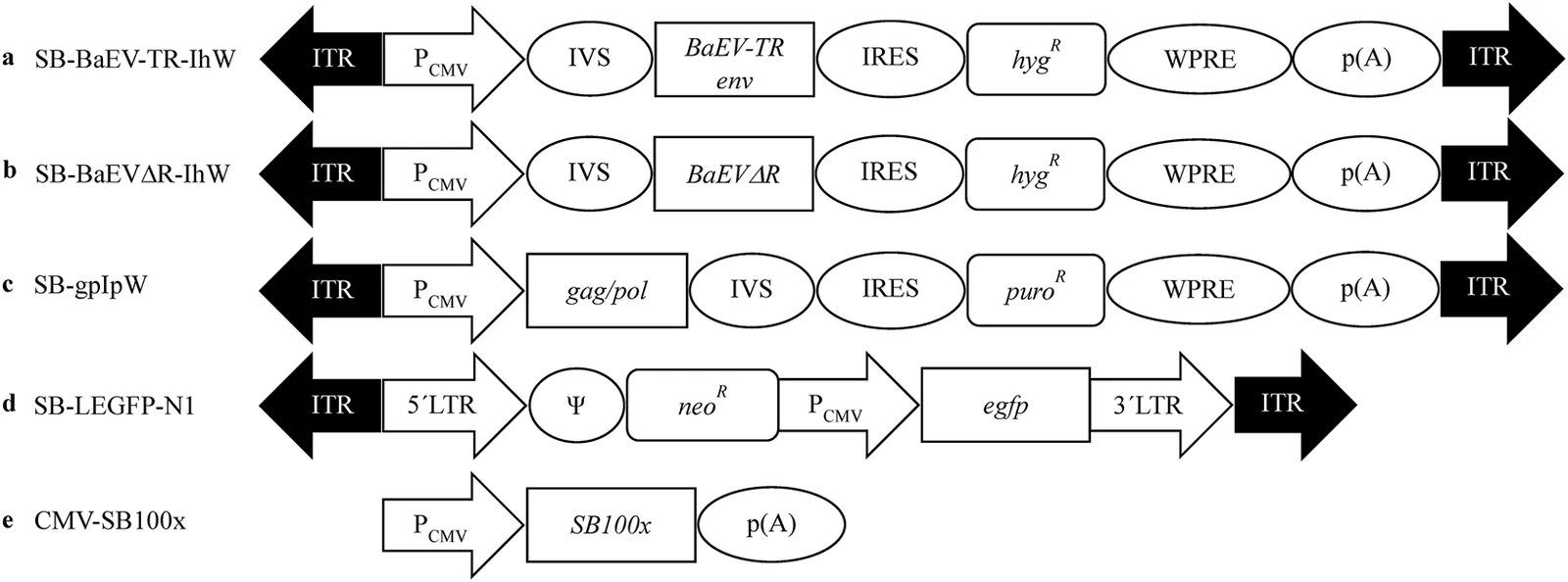
Schematic illustration of the constructs used for packaging cell line establishment. **(a)** The envelope construct SB-BaEV-TR-IhW encompasses a cytomegalovirus (CMV) promoter/enhancer element (P_CMV_), which drives the expression of the *BaEV-TR env* gene downstream of a synthetic intron (IVS), followed by an IRES mediating geno-phenotype coupling with the hygromycin-resistance gene (*hyg* ^*R*^). The woodchuck hepatitis virus post-transcriptional regulatory element (WPRE) and the polyadenylation signal (p**(a)**) of the bovine growth hormone gene are located at the 3′-end. In addition, Sleeping Beauty-derived ITRs flank the expression cassette. **(b)** The envelope construct SB-BaEVΔR-IhW encodes the R-peptide-deleted Env variant BaEVΔR, which is embedded in the same backbone construct as BaEV-TR Env. **(c)** The packaging construct SB-gpIpW encodes the *gag/pol* viral core proteins and enzymes as well as the puromycin-resistance gene (*puro* ^*R*^). **(d)** The transfer vector construct SB-LEGFP-N1 contains the MLV packaging signal ψ and the 5′- and 3′-LTRs harboring the MLV promoter, driving the expression of the neomycin-resistance gene (*neo* ^*R*^) and the transcription of the full-length transfer vector. Moreover, P_CMV_ facilitates the expression of the reporter gene *egfp*. **(e)** The transposase construct CMV-SB100X entails the human codon-optimized Sleeping Beauty transposase variant gene SB100x.

The packaging construct SB-gpIpW, the transfer vector SB-LEGFP-N1, and the transposase plasmid CMV-SB100x used in this study were previously described in detail (Berg et al., 2019). The BaEV-TR *env* DNA sequence (Girard-Gagnepain et al., 2014; GenBank accession no. NC_022517) was synthesized (GenScript, United States) and amplified using the oligonucleotides 5′-ACT​CCC​AGG​TCC​AAC​TGC​AGG​TCG​AGC​ATG​CAT​CTA​GGG​CGC​TAG​CAC​CAT​GGG​ACA​CAA​C-3′ and 5′-GAG​AGG​GGG​GGG​GGG​GAG​AGG​GGC​GGA​ATT​CCT​CTA​GTG​CCG​TAC​GT CATCATGGCTCGT-3´. The R-peptide-deleted *env* DNA sequence BaEVΔR was amplified using the oligonucleotides 5′-CTG​CAG​GTC​GAG​CAT​GCA​TCT​AGG​GCG​GCC​GCA​CCA​TGG​GAT​TCA​CA ACAAAG-3′ and 5′-GGG​GGA​GAG​GGG​CGG​AAT​TCC​TCT​AGT​GCT​ACA​TAG​CGT​GTA​TTA​T GT-3´. The amplicons were inserted using Gibson assembly (Gibson et al., 2009) into the SB-IhW backbone (Berg et al., 2019) linearized with NotI using the HiFi Master Mix (Thermo Fisher Scientific, Germany), resulting in the envelope constructs SB-BaEV-TR-IhW and SB-BaEVΔR-IhW, respectively. In these transposon vectors, envelope protein expression is driven by a strong promoter/enhancer of cytomegalovirus and coupled via an internal ribosome entry site (IRES) to a hygromycin resistance gene. The accuracy of all plasmids was confirmed using DNA sequencing. The genetic organization of the expression cassette components of all constructs employed in this study is schematically illustrated in Figure 1.

### 3.3 Syncytia formation assay

To assess whether envelope proteins mediated syncytia formation in transiently transfected HT1080 and 293T cells, 2.0 × 10^5^ and 4.0 × 10^5^ cells, respectively, and reflecting the different cell sizes, were seeded in a total volume of 2.5 mL in six-well dishes. Two hours post-seeding, 1.25 µg of an EGFP-encoding reporter construct together with either 1.25 µg of SB-BaEV-TR-IhW, the SB-BaEVΔR-IhW envelope construct serving as a positive control, or the SB-IhW backbone construct serving as a negative control (mock), were mixed in 250 µL of Opti-MEM (Gibco, Germany). A 7.5 µL aliquot of the transfection reagent TransIT-LT1 (Taurus, United States) was added, mixed, and incubated at room temperature for 20 min. Subsequently, the resulting DNA complexes were added to the cells. Four days post-transfection, cells were microscopically examined (Axio Vert A1, Zeiss, Germany).

### 3.4 Establishment of the stable VPCL HT/BaEV-TR

HT1080 cells were seeded at a density of 2.0 × 10^5^ cells per well in a six-well dish (Nunc, Germany) and, 2 hours later, transfected using the reagent TransIT-LT1 (Taurus, United States): 0.75 µg of both the envelope and packaging constructs, 1.5 µg of the transfer vector, together with 0.2 µg of the transposase construct, were transfected as described above. Three days post-transfection, cells were detached, transferred to T75 flasks (Nunc, Germany), and expanded in the presence of increasing concentrations of the three antibiotics G418 (Thermo Fisher Scientific, Germany), hygromycin B, and puromycin (InvivoGen, United States) as previously described (Berg et al., 2019).

### 3.5 Western blot analysis

A 2 mL aliquot of the cell-free supernatants of HT/BaEV-TR and naïve HT1080 was harvested and pelleted at 4 °C and 15,000 rpm for 1.5 h using a Micro 200R centrifuge (Hettich, Germany). Pellets were resuspended in 30 µL RIPA buffer (Thermo Scientific, Germany) and incubated for 30 min on ice. Subsequently, 10 µL of ROTI®Load 2 (Carl Roth, Germany) supplemented with 1 M dithiothreitol (DTT; Carl Roth, Germany) was added, and the samples were heated at 95 °C for 15 min. Equal volumes of the samples were loaded on a 10% Mini-PROTEAN® TGX™ Precast Protein Gel (Bio-Rad, United States) for SDS-PAGE and subsequent blotting on a nitrocellulose membrane (ROTI®NC 0.45; Carl Roth, Germany). The BaEV Env was detected using a polyclonal rabbit antibody specific for the extracellular domain of BaEV Env (GenScript, United States) at a dilution of 1:1,000. Thereafter, the membrane was incubated with secondary chicken anti-rabbit antibodies conjugated with horseradish peroxidase (HRP; 1:5,000; Abcam, United States). To detect MLV p30-CA, polyclonal goat antibodies (1:2,500; ATCC VR-1564AS-Gt) were utilized, followed by donkey anti-goat IgG HRP-conjugated secondary antibodies (1:2,000; Abcam, UK). To further improve the sensitivity of detection, recombinant HRP-coupled protein G (1:2,000; Invitrogen, United States) was added. The substrate ECLplus (Thermo Scientific, Germany) enabled luminescence visualization, and a ChemiDoc XRS + imager (Bio-Rad, United States) was employed for signal detection.

### 3.6 Viral vector titration

After reaching confluency, 10 mL of fresh media was added to the VPCL cultures. The supernatant was harvested after 24 h and passed through a 0.45 µm pore size PVDF filter (Carl Roth, Germany) to obtain cell-free vector preparations. To assess the viral vector titer, 2.0 × 10^5^ HT1080 and 4.0 × 10^5^ 293T adherent target cells were seeded in six-well dishes (CytoOne, Germany). Two hours post-seeding, different dilutions of vector preparations, namely, 1:10, 1:50, 1:100, and 1:200, were employed in transduction experiments in a total volume of 1 mL. Jurkat, U937, K562, and NB4 cells were subjected to spin-transduction. A total of 1.0 × 10^5^ cells were centrifuged at 3,300 rpm for 3 h at 30 °C in a total volume of 1 mL containing the vector dilutions. After resuspension, the cells were transferred to a 12-well dish (CytoOne, Germany). Two to 3 days post-transduction, cells were analyzed employing flow cytometry (SH800, Sony, Japan) to determine the percentage of EGFP-positive cells. Vector titers were calculated using cell-free supernatants of the VPCL in dilutions resulting in 1%–10% EGFP-expressing cells, applying the following formula (Salmon and Trono, 2007):

$$Titer\;\left[\frac{TU}{mL}\right]=number\;target\;cells\;x\;\left(\frac{\%\;of\;GFP\;positive\;cells}{100}\right)\;x\;dilution\;factor.$$

## 4 Results

### 4.1 BaEV-TR does not mediate syncytia formation in 293T and HT1080 cells

To assess the fusogenic activity of the envelope protein variant BaEV-TR and, thus, its utility to establish high-titer VPCLs, two suitable host cell lines were used in a syncytia formation assay. In addition to 293T, HT1080 human fibrosarcoma host cells are known to facilitate the establishment of high-titer MLV-based VPCLs using traditional plasmid transfection (Cosset et al., 1995) and transposon vector technology (Berg et al., 2019).

**FIGURE 2:**
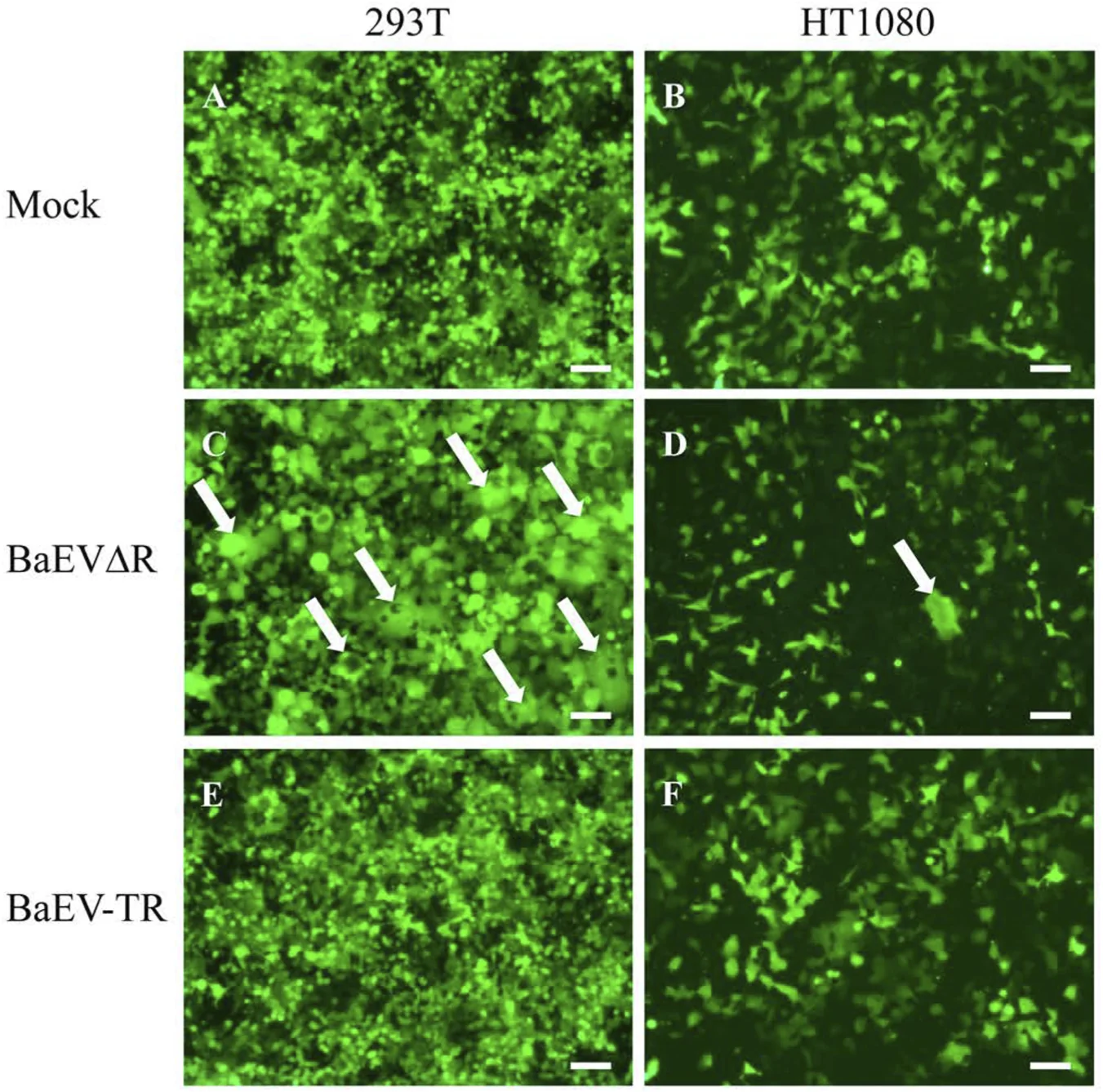
Fluorescence microscopic images of transiently transfected 293T and HT1080 cells. **(A,B)** 239T (left) and HT1080 cells (right) transfected with an EGFP-encoding reporter plasmid and SB-IhW serving as a negative control (mock). **(C,D)** Positive controls consisted of both cell lines transfected with the EGFP-coding reporter plasmid and the envelope construct SB-BaEVΔR-IhW. **(E,F)** Both cell populations transfected with the EGFP-coding reporter plasmid and SB-BaEV-TR-IhW. All images were taken 4 days post-transfection at 100-fold magnification. White arrows indicate syncytia. Scale bars represent 100 µm.

HT1080 and 293T cells were transiently co-transfected with an EGFP-encoding reporter construct and the respective expression construct. Cells co-transfected with the reporter construct in concert with the host vector SB-IhW, which did not encompass any *env* gene, served as negative controls. Positive control cells were co-transfected with the reporter and the construct SB-BaEVΔR-IhW encoding an R-peptide-truncated Env of BaEV. Comparable transfection efficiencies were visualized and confirmed by detecting EGFP using fluorescence microscopy. As illustrated in Figures 2A,B, no syncytia were observed in either cell population serving as negative controls over an extended cultivation period of 4 days. In contrast and as expected, numerous syncytia were observed in the 293T cell population expressing the BaEVΔR envelope proteins (Figure 2C). Noteworthy, syncytia were only rarely observed in the HT1080 cells expressing the R peptide-deleted Env (Figure 2D). No syncytia formation was observed in either of the cell lines transfected with the reporter plasmid and SB-BaEV-TR-IhW (Figures 2E,F). Apart from the described syncytia formation and the conduction of bright-field microscopic inspections, no morphological changes were observed in any of the confluent cell populations over the entire 4-day period.

### 4.2 BaEV-TR expression does not impede the establishment of a stable VPCL

As no syncytia mediated by BaEV-TR proteins and only very few syncytia upon BaEVΔR expression were observed in HT1080 human fibrosarcoma host cells, this cell line was selected to establish a stable VPCL using the Env variant BaEV-TR. HT1080 cells were consequently co-transfected with the Sleeping Beauty transposon-derived expression constructs illustrated in Figure 1, namely, SB-BaEV-TR-IhW, the packaging construct SB-gpIpW, the transfer vector construct SB-LEGFP-N1, and the transposase plasmid CMV-SB100x. The stable polyclonal VPCL HT/BaEV-TR was obtained upon subsequent selection with increasing concentrations of antibiotics, as previously described (Berg et al., 2019). Upon a selection period of only 3 weeks, the final antibiotic concentrations of 3 μg/mL puromycin, 300 μg/mL hygromycin, and 400 μg/mL G418 were reached, resulting in the polyclonal VPCL termed HT/BaEV-TR.

### 4.3 BaEV-TR Env are readily detected in MLV vector particles

**FIGURE 3:**
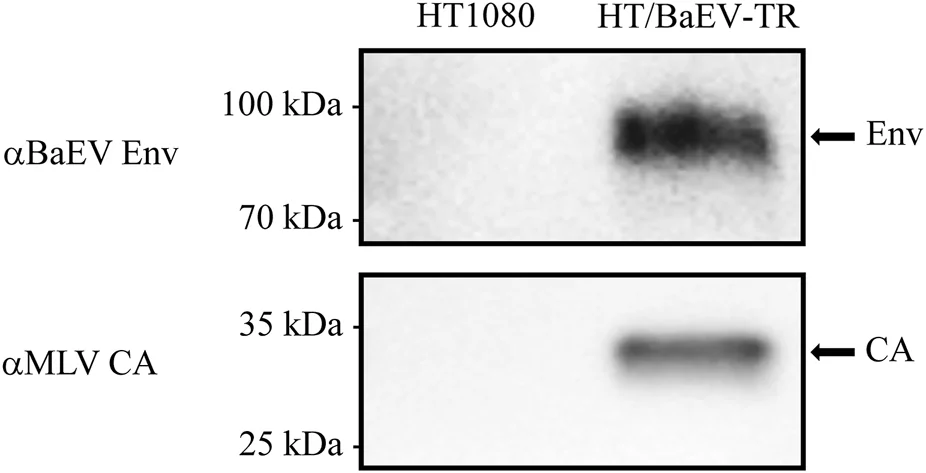
Incorporation of BaEV-TR Env proteins in MLV vector particles. Cell-free supernatants of HT/BaEV-TR packaging cells and naïve HT1080 cells serving as negative controls were pelleted at 15,000 rpm. Western blot analysis of vector particle pellets was performed using polyclonal rabbit antibodies directed against the extracellular domain of the precursor Env protein of BaEV and polyclonal goat antibodies against MLV capsid (CA) protein p30. The positions of the molecular weight markers are indicated on the left.

To assess the incorporation of BaEV-TR Env into MLV-based vector particles, cell-free supernatants of the stable VPCL HT/BaEV-TR were collected and subjected to centrifugation at 15,000 rpm using a benchtop centrifuge. In a Western blot analysis, the resulting pellets were analyzed using antibodies specific for the extracellular domain of BaEV Env and MLV p30-CA capsid proteins, followed by secondary antibodies coupled to horseradish peroxidase (HRP). A sample from naïve HT1080 served as a negative control. As depicted in Figure 3, MLV CA and BaEV Env proteins were readily detected in the sample from the VPCL but not in the negative control. This result supported the expected incorporation of BaEV-TR envelope proteins into MLV-derived vector particles.

### 4.4 MLV(BaEV-TR) pseudotype vectors efficiently transduce a variety of human target cell lines

To examine the transduction efficiency of the pseudotype vectors produced by the VPCL HT/BaEV-TR, cell-free vector particle preparations from different cell passages at approximately 1-week intervals were harvested and subjected to transduction experiments. Human target cells included adherent 293T and HT1080 cells, Jurkat T cells, pro-monocytic U937, K562 lymphoblasts, and acute promyelocytic leukemia NB4 cells. Untransduced cells served as negative controls. Two to three days post-transduction, FACS analysis was performed to detect EGFP-positive cells, revealing high gene transfer efficiencies mediated by MLV(BaEV-TR) pseudotype γ-retroviral vector particles in three independent experiments. As reported in Table 1, the mean vector titer obtained in 293T cells reached 3.3 × 10^6^ transducing units per mL (TU/mL) and was notably higher than that obtained in HT1080 cells, which yielded 4.8 × 10^5^ TU/mL. Mean titer values in human hematopoietic cell lines ranged from 6.1 × 10^5^ TU/mL in K562, 5.2 × 10^5^ TU/mL in Jurkat, 8.0 × 10^5^ TU/mL in U937, and 1.0 × 10^5^ TU/mL in NB4 cells.

**TABLE 1:** MLV (BaEV-TR) pseudotype vector titers obtained from three independent titration experiments using different human target cell lines. Human embryonic kidney 293T cells, fibrosarcoma HT1080 cells, Jurkat T cells, pro-monocytic U937, K562 lymphoblasts, and acute promyelocytic leukemia NB4 cells served as target cell lines. Titers are reported as transducing units per mL (TU/mL). Mean values from titrations with vector preparations from different passages in weekly intervals are shown with respective standard deviations (SD). A standard static transduction was conducted with adherent 293T and HT1080 target cells; suspension cell lines (Jurkat, U937, K562, and NB4) were subjected to spin-transduction.

Target cell line	Titer [TU/mL]			Mean ± SD
	Exp. 1	Exp. 2	Exp. 3	
293 T	4.2 × 10^6^	3.0 × 10^6^	2.8 × 10^6^	3.3 × 10^6^ ± 6.2 × 10^5^
HT1080	4.3 × 10^5^	4.1 × 10^5^	5.9 × 10^5^	4.8 × 10^5^ ± 8.1 × 10^4^
Jurkat	4.3 × 10^5^	6.4 × 10^5^	5.0 × 10^5^	5.2 × 10^5^ ± 8.4 × 10^4^
U937	8.8 × 10^5^	8.9 × 10^5^	6.3 × 10^5^	8.0 × 10^5^ ± 1.2 × 10^5^
K562	7.9 × 10^5^	4.0 × 10^5^	6.4 × 10^5^	6.1 × 10^5^ ± 1.6 × 10^5^
NB4	1.6 × 10^5^	7.4 × 10^4^	7.0 × 10^4^	1.0 × 10^5^ ± 4.2 × 10^4^

## 5 Discussion

MLV-derived pseudotyped vectors are frequently used in a variety of gene therapeutic strategies, mostly aiming at the genetic modification of hematopoietic cell lines. To improve gene transfer efficiencies, Env proteins from a range of different donor viruses have been used successfully, recruiting cognate cellular receptors expressed on the target cell type of choice. The envelope proteins of BaEV mediated highly efficient gene transduction into primary human blood cell types when MLV (BaEV) pseudotype vectors were produced by transiently transfected 293T cells (Ciulean et al., 2023). However, when constitutively expressed in 293T cells, BaEV utilized the receptors ASCT1 and ASCT2, the knockout of which was required to enable stable VPCL generation using wt BaEV and R-peptide-deleted Env proteins (Zhao et al., 2025), rendering the establishment of novel cell lines cumbersome and time-intensive.

The Env variant BaEV-TR, encompassing the cytoplasmic tail and R-peptide (TR) of MLV, was previously reported not to mediate cell-to-cell fusion resulting in syncytia formation in transiently transfected 293T cells to produce lentiviral vectors (Girard-Gagnepain et al., 2014). Env-TR variants of other parental viruses facilitate improved pseudotype MLV vector particle formation, yielding higher titers compared to the wt Env proteins (Lindemann et al., 1997; Stitz et al., 2000; Sandrin and Cosset, 2006), most likely capitalizing on the specific interaction of the MLV R-peptide with Gag core proteins (Rein et al., 1994; Schneider et al., 2011). To assess the utility of BaEV-TR proteins in the establishment of human host cell lines without receptor knockout, we first examined the absence of syncytia formation in 293T and HT1080 cells in a syncytia formation assay. In addition to the most frequently used 293 derivate host cells, human fibrosarcoma HT1080 cells are known to support high-yield pseudotype vector production (Cosset et al., 1995). No syncytia were observed in either of the two cell lines transiently expressing BaEV-TR proteins, confirming the absence of cytotoxicity via syncytia formation. In contrast and as expected, BaEVΔR Env mediated the formation of syncytia in both cell lines, proving high fusogenic activity. However, in HT1080 cells, syncytia were observed at a much lower frequency compared to 293T cells. This hints at a comparatively lower receptor density on the fibrosarcoma cells. However, to verify this hypothesis, examination of quantitative receptor expression levels would be required.

We thus chose HT1080 cells for the establishment of the stable VPCL as a low receptor expression level should provide two advantages. i) The cell surface localization of the envelope proteins should be less hampered by premature interaction with the receptor already during cellular expression and transport in the endoplasmic reticulum, Golgi apparatus, and vesicles in the VPCL. Thus, more Env should be available for incorporation into vector particles. ii) Receptor interference, i.e., resistance to superinfection (Sommerfelt and Weiss, 1990), should lead to a lowered risk of re-transduction as only very few receptor molecules are available to enable vector entry into VPCLs (Labenski et al., 2016). Moreover, the availability of receptors should be further lowered by the shedding of the surface unit of Env and the subsequent competition for receptor recruitment by MLV (BaEV) pseudotype particles.

Using transposon vector technology and subsequent increasingly stringent selection, the stable polyclonal VPCL HT/BaEV-TR was successfully established. As expected and in accordance with Env-TR variants derived from other donor viruses mentioned above, Western blot analysis of vector particles employing antibodies specific for the extracellular domains of BaEV Env proteins revealed incorporation of the heterologous chimeric envelopes into MLV-based vector particles. In addition, transduction experiments using cell-free supernatants (CFSNs) of the polyclonal HT/BaEV-TR VPCL and a panel of human target cell lines were performed. Over an extended period of a few months, high vector titers were repeatably observed, indicating stable productivity, as previously reported for VPCLs using similar expression constructs (van Heuvel et al., 2023). In naïve adherent 293T and HT1080 cells, mean titers of 3.3 × 10^6^ TU/mL and 4.8 × 10^5^ TU/mL, respectively, were achieved. This further supports the assumption of a comparatively lower receptor density on HT1080 rather than on 293T cells. As MLV vectors can only transduce dividing cells, the proliferation rate or doubling time is another factor influencing vector titers in different target cells, clearly favoring 293T over HT1080 cells. It also cannot be excluded that retroviral restriction factors might limit transduction efficiencies in some target cell lines. Possibly most importantly, mean vector titers of 6.1 × 10^5^ TU/mL, 5.2 × 10^5^ TU/mL, 8.0 × 10^5^ TU/mL, and 1.0 × 10^5^ TU/mL in K562 lymphoblasts, Jurkat T lymphoblasts, pro-monocytic U937 cells, and acute promyelocytic leukemia NB4 cells, respectively, confirmed the BaEV-TR envelope variant as a potent mediator of γ-retroviral vector gene transfer into hematopoietic cell types. However, only established cell lines were utilized for transduction experiments, and validation in primary hematopoietic cells will be required in future studies.

Taken together, MLV (BaEV-TR) pseudotyped vectors can be produced continuously at high titers in stable HT1080 host cell-derived VPCLs and should thus prove their utility in future gene and cell therapeutic strategies aiming at the genetic modification of clinically relevant primary human cell types such as hematopoietic stem and progenitor cells, NK, B, and T cells (Girard-Gagnepain et al., 2014; Levy et al., 2016; Costa et al., 2017; Radek et al., 2019; Ciulean et al., 2023; Zhao et al., 2025; Périan et al., 2026; Pinot et al., 2025).

## Data Availability

The raw data supporting the conclusions of this article will be made available by the authors, without undue reservation.
